# An Experimental Framework for Generating Evolvable Chemical Systems in the Laboratory

**DOI:** 10.1007/s11084-016-9526-x

**Published:** 2016-11-18

**Authors:** David A. Baum, Kalin Vetsigian

**Affiliations:** 1 Department of Botany, University of Wisconsin, 430 Lincoln Drive, Madison, WI 53706 USA; 2 Wisconsin Institute for Discovery, University of Wisconsin, 330 N. Orchard Street, Madison, WI 53706 USA; 3 Department of Bacteriology, University of Wisconsin, 1550 Linden Drive, Madison, WI 53706 USA

**Keywords:** Autocatalysis, Ecosystem selection, Life-like chemistry, Neighborhood selection, Prebiotic chemistry, Self-propagation, Surface catalysis

## Abstract

Most experimental work on the origin of life has focused on either characterizing the chemical synthesis of particular biochemicals and their precursors or on *designing* simple chemical systems that manifest life-like properties such as self-propagation or adaptive evolution. Here we propose a new class of experiments, analogous to artificial ecosystem selection, where we *select* for spontaneously forming self-propagating chemical assemblages in the lab and then seek evidence of a response to that selection as a key indicator that life-like chemical systems have arisen. Since surfaces and surface metabolism likely played an important role in the origin of life, a key experimental challenge is to find conditions that foster nucleation and spread of chemical consortia on surfaces. We propose high-throughput screening of a diverse set of conditions in order to identify combinations of “food,” energy sources, and mineral surfaces that foster the emergence of surface-associated chemical consortia that are capable of adaptive evolution. Identification of such systems would greatly advance our understanding of the emergence of self-propagating entities and the onset of adaptive evolution during the origin of life.

## Introduction

The core of the origin of life problem is to explain the emergence of chemical systems that exhibit the capacity for heritable change and open-ended evolution. Once such systems arise, adaptive evolution can take over and, given sufficient time, explain the complexity and diversity of life as we know it. Even if we assume that the first step was the spontaneous appearance of evolvable, autocatalytic chemical systems, many unanswered questions remain. What factors must be present for the emergence of systems that show collective multiplication of all members of the system? When such systems do arise, are they necessarily capable of evolution? And, if evolvable, is their chemical make-up predictable, such that the same chemistry would tend to arise repeatedly, or would each independent origin be chemically distinct? Answering these questions is important not only for understanding the origin of life on Earth but also for evaluating the probability of life, broadly understood, elsewhere in the universe.

The best way to study the rules governing the emergence of life would be to establish conditions in the laboratory in which we could observe the repeated de novo emergence of life-like chemistry, which is to say self-propagating chemical systems capable of adaptive evolution. (We use the term “self-propagation” rather than “self-replication” to better describe systems that lack clear boundaries yet show collective multiplication of all members of the system.) This may seem ambitious in the extreme. However, we will argue in this paper that life-like chemical systems may arise quickly in some conditions and, consequently, the key challenge is not generating them but detecting them when and if they arise. Further, we suggest that the most promising general approach to detecting de novo life-like systems is based on their capacity for adaptive evolution. Based on these insights, we propose a new class of experiments that impose selection on abiotic chemical systems and search for situations in which an adaptive response arises. While emergence of systems capable of extensive evolution within the laboratory is unlikely, identifying conditions in which self-propagation and a few adaptive transitions can be observed would still be of great value. Given the relative simplicity of this approach, which sits at the intersection of synthetic and universal approaches to the origin of life (Scharf et al. 2015), we suggest that conducting prebiotic selection experiments should be a priority for the origin of life community.

Our approach is conceptually similar to artificial ecosystem-level selection, in which one establishes a large number of ecosystems, each containing a diverse (usually microbial) community and then decides based on a measurable, ecosystem-level trait which ecosystems to use to establish the next generation of ecosystems (Swenson et al. 2000b). Computer simulations have demonstrated the potential for ecosystem-level selection (Penn and Harvey 2004; Williams and Lenton 2007), and several experiments with microbial microcosms have shown that at least some response to ecosystem selection is possible and that the associated heritability might be due to changes in community states rather than genetic changes of individual species (Swenson et al. 2000a; Day et al. 2011; Blouin et al. 2015). These studies highlight that new levels of selection can emerge from the dynamics of systems of interacting entities, and thus give hope that the emergence of units of selection will also be possible in communities of interacting molecules. Of course there are important differences between ecological dynamics and molecular assemblages. In particular, whereas new organisms arise from existing organisms, new molecules typically do not self-replicate but arise from reactions among other molecular species. At the very minimum, ecosystem-level selection demonstrates the potential power of in vitro selection to detect emergent levels of organization, making it an approach that has something to offer to the origin of life field.

## Background and Principles

A modern cell can be viewed as a heterogeneous consortium of organic and inorganic chemicals that together have the ability, in certain permissive environments, to assimilate or synthesize more of the same kinds of chemicals in roughly the same proportions. Take a cell in a suitable environment and it will double in volume: for every one chemical moiety of a particular kind in the starting cell, there eventually come to be approximately two. This capacity for growth resides nowhere other than in the chemicals that make up the cell (or at least a large subset thereof). As long as all of these critical chemicals remain present at approximately fixed stoichiometry, the capacity for growth in the same environmental conditions will perpetuate itself indefinitely.

Generalizing from the case of cells, we can consider chemical assemblages to be life-like insofar as they have the potential to cause their own growth (collective multiplication) in a permissive environment. It might seem that the ability to grow is an all or nothing property that draws a sharp dividing line between life and non-life. Notice, however, that the growth of cells can vary in its fidelity (maintenance of stoichiometric parity), tolerance (range of environments in which doubling is possible), or efficiency (time or energy used per doubling). From this, we can imagine the existence of chemical assemblages that manifest life-like chemistry but are much less faithful, tolerant, or efficient than living cells. To see this gradation, let us work down from cells to protocells and then to hypothetical surface-bound chemical assemblages.

Life-like chemistry is not restricted to modern cells with template-based inheritance systems (Segrè et al. 2000; Shenhav et al. 2003; Markovitch and Lancet 2012). A membrane-bound vesicle that contained a set of cooperating chemicals that could assimilate or synthesize more of the same chemicals would be life-like. Like true cells, such an entity would have the potential to grow, divide, and evolve by selection. In such mesobiotic entities (sensu Shenhav et al. 2003), heredity still applies despite the lack of a digital genetic information-encoding system. A mesobiotic protocell shows analog inheritance in the sense that its genotype is a “compositional genome” (Segrè et al. 2000; Markovitch and Lancet 2012) corresponding to the stoichiometry of its component chemical moieties. While these systems would have low heritability if governed by pure stoichiometry (Vasas et al. 2010), as dynamical autocatalytic systems they can manifest homeostatic properties in the vicinity of attractor states (Markovitch and Lancet 2012). As a result, it is plausible that mesobiotic entities can evolve by natural selection when occasional stochastic perturbations move the system to a new quasistable state that confers higher fitness (Markovitch and Lancet 2012). Thus, a mesobiotic protocell with fully analog inheritance could nonetheless evolve adaptively towards higher fidelity, tolerance, and efficiency. This implies that digital genetic encoding might not have been present at the onset of adaptive evolution but arose at a later date when RNA-catalyzed peptide synthesis came to be controlled by a guide mRNA sequence. Given the complexity of the translation machinery, it certainly seems more plausible that guided translation arose via a pre-existing selective process than that it arose spontaneously without prior adaptive evolution.

By focusing on cells and protocells, the foregoing discussion has been restricted to evolvable entities with sharp boundaries. However, growth and evolution of molecular assemblages does not inherently depend upon such boundedness. In principle, such phenomena should also be possible in spatially structured environments, such as solid surfaces in which molecules can maintain prolonged associations (Takeuchi and Hogeweg 2009; Virgo et al. 2009; Froese et al. 2014). Given the abundance of mineral surfaces in the prebiotic world, growth and spread of life-like chemistry on surfaces has been proposed as a more plausible alternative to the spontaneous appearance of evolving protocells or compartments (Wächtershäuser 1988; Cleaves et al. 2012; Wu and Higgs 2012; Baum 2015). Chemical mixtures adsorbed onto such surfaces could cooperate in autocatalytic systems to cause local enrichment of members of these autocatalytic sets. Furthermore, insofar as there is spatial heterogeneity in the adsorbed chemicals, areas that are occupied by more effective (fitter) autocatalytic systems will tend to predominate.

Given this perspective, if a barely life-like assemblage arose on a mineral surface and the environment remained favorable, including consistent replenishment of chemical precursors (“food”) and appropriate energy to overcome entropic decay, we should expect the assemblage to evolve by a variant of natural selection called “neighborhood selection” (Nunney 1985; Baum 2015). Such ensembles would tend to become better at growing over the surface, whether by displacement of adjacent neighborhoods or rapid colonization of newly exposed mineral surfaces. Neighborhood selection might act to favor innovations that arise. For example, rare/improbable side reactions might allow the system to move to a new quasistable state of higher complexity. Eventually such selection would likely favor the production of propagules, lipid bound vesicles including a sample of the surface protoplasm, whose original role was to colonize new mineral surfaces, but which eventually evolved into protocells capable of growing and dividing free of a mineral surface (Baum 2015).

The details of neighborhood selection certainly deserve rigorous theoretical analysis, but available models give grounds for believing that the framework is reasonable. Two-dimensional, spatial models reveal that self-catalytic structures can be resistant to molecular parasites, and mutualisms between replicating structures can be promoted, leading to adaptive evolution towards higher fidelity and speed of growth (Szabó et al. 2002; Hogeweg and Takeuchi 2003; Könnyű et al. 2008; Takeuchi and Hogeweg 2009). Indeed, counter-intuitively, some superficially parasitic side reactions can promote persistence of autocatalytic systems by generating motility over a surface (Virgo et al. 2013). Cooperation is especially promoted if it involves coupling through production of metabolic precursors (Könnyű and Czárán 2015; Könnyű et al. 2008; Czárán et al. 2015) or if growth proceeds through spatial expansions into unoccupied regions (Van Dyken et al. 2013; Shay et al. 2015). It is also noteworthy that surfaces may promote multi-step reactions that would be improbable in solution (Bauler et al. 2010; Cleaves et al. 2012; Castellana et al. 2014), and that certain mineral surfaces can participate directly in the synthesis of polymers (Wächtershäuser 1988, 1990, 2007; Huber and Wächtershäuser 1998; Ferris et al. 2006; Hazen and Sverjensky 2010). For all these reasons it is reasonable to assume that an early step in the origin of life entailed the evolution of chemical systems adsorbed onto mineral surfaces, raising the potential value of studying such systems in the laboratory.

## The Plausibility of Studying Life-like Chemical Systems in the Laboratory

Before embarking on laboratory experiments, a basic question is whether entities capable of adaptive evolution can arise generically in a wide range of suitable contexts or if they require exceedingly rare combinations of circumstances. If the latter were the case, then the onset of adaptive evolution would be fundamentally unobservable on the spatial and temporal scales of laboratory science. We will now argue, however, that nothing that we know from physics, geology, chemistry or mathematical modeling of autocatalytic systems precludes the possibility that de novo systems capable of adaptive evolution might arise with high probability given suitable contexts.

It is well appreciated that dissipative structures can readily arise in systems far from thermodynamic equilibrium and that life itself is a dissipative structure. It is possible to observe spontaneous emergence of such structures in many situations [e.g., tornadoes, snowflakes, chemical oscillators, actin filaments and microtubules (Boekhoven et al. 2015)], but to date they are fixed in their properties and therefore cannot adapt. It can be argued, however, that this state of affairs is a consequence of the simplicity of the initial and boundary conditions (Ikegami and Hanczyc 2009; Armstrong 2015). If the initial conditions were complex, for example containing a great diversity of different chemicals, they might readily yield autocatalytic dynamics, even in the absence of high-specificity catalysts (Virgo and Ikegami 2013). As a result, multiple different dissipative structures are likely to emerge, and interactions between them can lead to self-organization at even higher levels (Melamede 2008; Pascal et al. 2013). This model is consistent with theoretical work on far-from-equilibrium systems (Pross and Pascal 2013; England 2015; Pascal and Pross 2015) and suggests the possibility that chemical dissipative structures possessing some level of adaptability might readily arise. The question may not be, Can evolvable chemical systems arise spontaneously in the lab? but, How can we experimentally promote the appearance of such systems and then detect them when they arise?

The geological record also does not reject the possibility that life on earth arose rapidly, since evidence of life has now been reported in several of the oldest known rocks (Mojzsis et al. 1996; Bowring and Williams 1999; McKeegan et al. 2007; Bell et al. 2015; Nutman et al. 2016). Furthermore, even if we suppose that there was a substantial gap between the formation of the earth and the appearance of life, some of this would likely reflect the need for a prolonged period of geological and chemical evolution to accumulate the necessary molecular building blocks of life. Given that this early chemical phase can be circumvented in the laboratory by starting systems with enriched chemical mixtures, the geological record is, if anything, quite encouraging for the prospective experimentalist.

Another source of historical evidence could come by looking around us and counting-up the number of origins of life that have occurred on earth. There are so many detailed and unexpected features that are shared by all known life forms that it is quite clear that they all share some degree of common ancestry. At first glance, the unity of life suggests the difficulty of life emerging: If life were easy then shouldn’t there be many flavors of life around us? However, this logic is flawed. First, it should be born in mind that even if there existed one cell that was ancestral to all extant life, that ancestral cell could represent a fusion or merger of multiple, independently-evolved earlier lineages. Second, as first argued by Darwin himself (letter to J. D. Hooker, April 17, 1863), the abundance of complex life everywhere on the planet would mitigate against the emergence of any de novo organic life forms because such new life might be quickly devoured. Thus, new life could have emerged constantly and repeatedly, but once sophisticated cellular life arose it pre-empted new life ever again getting to a level of complexity that could easily be detected. Finally, it cannot be ruled out that other very simple life forms do exist on Earth, since it is very unclear how we could detect it. Thus, the universal common ancestry of known cellular life is neither here nor there for assessing the likely speed and ease with which life-life chemical systems can emerge.

Mathematical modeling combined with basic chemical knowledge is another way to assess the plausibility of new evolvable systems emerging. Models that assume the appearance of a single heteropolymer, such as RNA, that can self-replicate through template-directed synthesis, while simple, do not support a rapid or easy onset of adaptive evolution. This is because, a self-replicating molecule would need to arise whose mutation rate was below the error catastrophe threshold (Eigen and Schuster 1977, 1978), which is very improbable (Maynard Smith 1979). Indeed, despite an intensive effort, no one has managed to design or select for a self-replicating RNA with anything close to sufficient fidelity (Joyce 2007). However, much greater optimism is possible if one imagines that life began as an autocatalytic chemical system (ACS) that consisted of multiple molecules (perhaps, but not necessarily, including RNAs) that showed collective multiplication (Kauffman 1986; Shenhav et al. 2003; Mossel and Steel 2005; Plasson et al. 2011). As discussed earlier, an ACS can evolve via changes in the stoichiometry of its components (Shenhav et al. 2003; Markovitch and Lancet 2012) or by the addition of reactions to the maximal ACS, for example via cooperation between ACSs with overlapping members (Hordijk et al. 2012). Combined with results showing that spatial structure, such as that provided by solid surfaces, can promote the emergence and persistence of ACSs (see Introduction), theory allows that it may be possible to observe the emergence of self-propagating and evolvable chemical systems in the laboratory. Indeed, given these results, a case can be made that an adsorptive surface fed a constant supply of diverse organic building blocks and provided with an appropriate source of free energy might rapidly become colonized by life-like chemical systems. But, how rapidly is “rapidly?” While we do not know for sure, there are no principles or results that would rule out the possibility of new life-like systems emerging and evolving adaptively within the time-frame of experimental laboratory science. The only way forward is to try.

## Deploying in Vitro Chemical Selection to Detect Life-like Chemical Systems

### General Concept

Suppose we could establish conditions under which simple life-like chemical assemblages would arise spontaneously on mineral surfaces and then evolve towards greater complexity by neighborhood selection. How would we know we had succeeded? A traditional approach might be to search for certain signatures that are taken to be diagnostic of life, for example RNA or proteins or bounded cells. However, there is no a priori reason to assume that de novo life-like chemical systems would necessarily bear these signatures. Furthermore, even if we believe that they would (or force the case by requiring that they *must* do so to be considered life-like), it might take millennia for these relatively complex signatures to arise. What we need is a signature of life-like chemistry that would apply to even the very simplest life-like system while also being relatively agnostic as to the chemistry involved.

We propose that the ideal way to detect new life-like chemical systems is by detecting their ability to evolve adaptively. Finding that chemical systems evolve adaptively would suggest they are life-like even in complete ignorance of their actual chemistry. How, then, can we detect adaptive evolution?

**Fig. 1:**
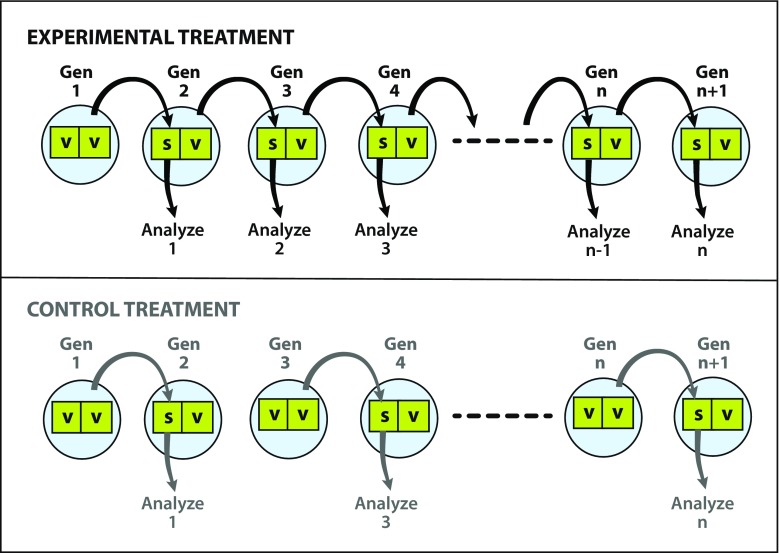
Basic experimental set-up. In the experimental treatment (*top*), a virgin wafer (v) is added to a seed wafer (s) from a previous generation. After one generation of incubation with a seed wafer, a virgin wafer becomes a seed wafer in the sense that, if a surface-associated chemical system had arisen that could colonize new surfaces, the former virgin wafer might now carry this self-propagating chemical system. In the control experiment (*bottom*), seed wafers are not carried forward to future generations, which should prevent progressive evolution of a life-like chemical system. As a result, consistent differences between the experimental and control treatments in later generations (e.g., generation n) would indicate that a self-propagating chemical system had arisen and been passed down the generations on sequential seed wafers

One possible approach is to select for the ability of a chemical system to colonize new surfaces and then look for changes in the rate of surface colonization. A simple way to impose such selection would be to establish a rotation in which old wafers (“seed” wafers), which might have been colonized by an ACS, are moved into fresh reagents with a virgin (i.e., uncolonized) wafer, but then the former virgin wafer is used as a seed for the next generation (Fig. 1). If a chemical system capable of surface colonization had arisen on the old wafer, it could seed the colonization of the virgin wafer and thereby ensure continued self-propagation. Furthermore, if there were a later transition that resulted in the chemical system showing more efficient colonization of available mineral surfaces, this new state would be favored and would tend to take over.

After multiple generations (sequential replacements of a previously exposed wafer with a virgin wafer) it would be possible to evaluate whether there had been any systematic, directional changes that would indicate a response to selection. Insofar as wafers from early and late generations are exposed to the same food/energy mix for the same amount of time, we should only see systematic changes over generations if systems have arisen that can be passed from wafer to wafer. In the absence of such hitchhiking, the first wafer removed from the chamber and the one-hundredth should be statistically indistinguishable.

What is attractive about this approach is that progressive changes in *any* physical or chemical feature of the system would suggest that a life-like chemical system had emerged. If we can detect changes in the rate at which virgin surfaces are colonized using a proxy trait that is relatively agnostic on the chemistry that is doing the colonization (see below) then we have a powerful way of documenting the emergence of evolvable chemical systems.

**Fig. 2:**
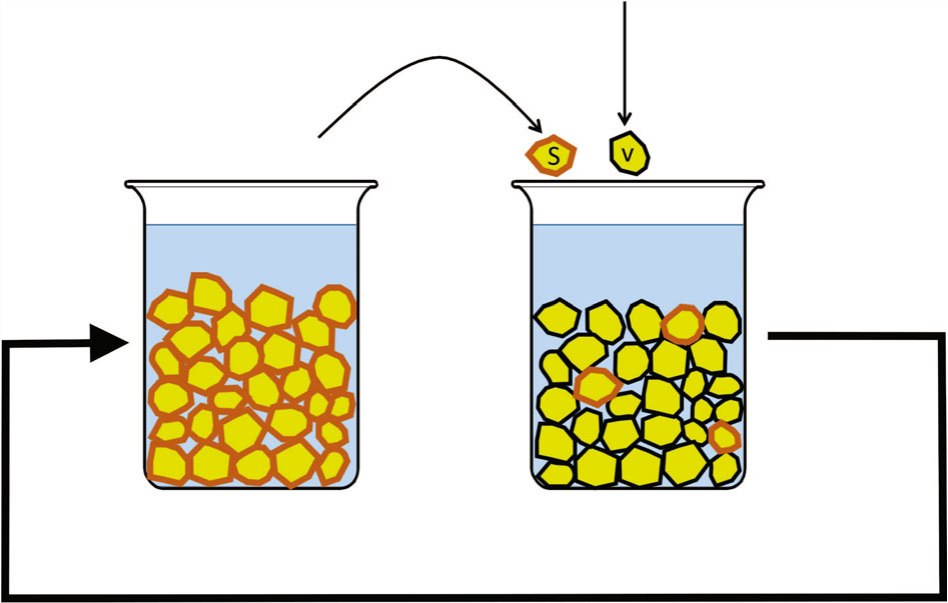
Experimental design using particles. After a period of incubation, during which particles may be colonized by life-like chemical assemblages, a subset of seed particles (with their adsorbed chemicals), *s*, would be transferred to a new container containing virgin particles, *v*, and a new food and energy solution. This experiment is conceptually similar to the wafer experiment (Fig. 1) but with a larger surface area and the ability to finely control the ratio of seed to virgin particles

Evolutionary theory tells us that maximal response to selection is achieved when population sizes are large. Population size is a less than obvious concept in the case of assemblages adsorbed onto a single continuous surface, but clearly the higher the surface area of mineral available, the larger the population. This suggests that prebiotic selection experiments might prove more successful if we used many small mineral crystals or grains rather than two large wafers. For example, we can submerge millions of mineral grains into a liquid food and energy mix and then transfer a small sample of “seed” particles to a new container containing virgin grains and fresh food mix (Fig. 2). As with the two-wafer experiment, the serial transfers should favor chemical systems that can grow on the surface of an individual grain and then move efficiently to other grains, meaning that chemical systems that were better at colonization would tend to be enriched over multiple generations. However, by dramatically increasing the surface area, this experiment might yield progressive changes that would not show up in the two-wafer case. Furthermore, if the particles were small enough to be transferred by pipetting, a particle experiment is amenable to an automated, high-throughput implementation.

Whether using wafers or particles, there are multiple features that could be monitored as evidence of changes in colonization rate. We could examine the chemical composition of the liquid phase through mass-spectrometry or track it colorimetrically to measure a single property such as pH or redox state. If the rate at which the food solution was altered within a generation changed, this would imply that adsorbed systems have evolved. Alternatively, the surface-associated material itself could be tracked. For example, we could use Energy Dispersive Spectroscopy to see if the amount of carbon or other elements accumulating on the surface in the course of a single generation changes over generations. Likewise, the relative abundance and spatial distribution of absorbed chemicals could be explored in more detail using ambient mass spectrometry techniques. Real-time analyses are also possible and could strengthen the case for life-like chemistry. For example, it might be possible to use thermal imaging to track heat production on surfaces as a proxy for energy dissipation, with the prediction that surfaces from later generations of selection will release more heat during incubation with the food and energy mix. Through these and similar analytical techniques it might be straightforward to see if there have been generation-by-generation changes in the assemblages accumulating on mineral surfaces.

### Interpretation of the Results

**Fig. 3:**
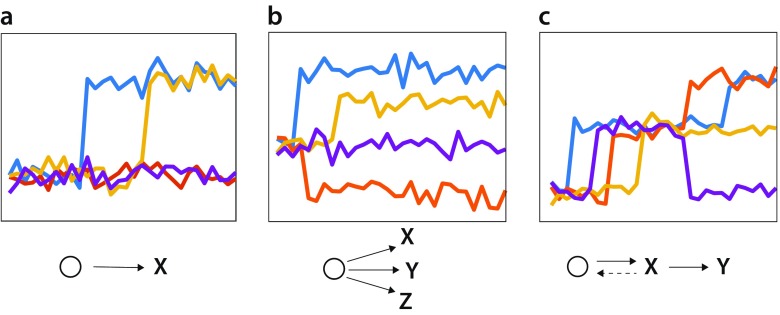
Sample results and their interpretation. Each graph shows hypothetical changes in a proxy trait (vertical axis) as a function of generation (horizontal axis) for four replicate experiments. For simplicity, we assume that control experiments, which are not shown, remain at the starting value for the proxy trait throughout the experiment. (**a**) Two of the replicates experience the emergence of the same self-propagating chemical system (X). (**b**) Whereas one replicate (*purple*) shows no significant change, the other three each transition to distinct self-propagating chemical systems (X, Y, Z), indicated by differing equilibrium values of the proxy trait. (**c**) Whereas all four replicates initially experience the emergence of the same self-propagating chemical system (X), this goes extinct in one replicate (*purple*) and evolves into a new chemical system (Y) in two others (*orange* and *blue*)

Trans-generational changes in physical or chemical features could be analyzed to infer the existence of chemical systems possessing life-like properties. There are two distinct phenomena that could be documented based on quantitative analysis of replicate wafer or particle experiments: (1) self-propagation, and (2) progressive evolution. To help explore these concepts, Fig. 3 shows some hypothetical results of tracking a suitable proxy trait (e.g., energy dissipation rate or rate of carbon accumulation) over multiple generations. Not shown on these figures are negative control runs, which would comprise new wafers/particles incubated in a given generation alongside the main experiments but lacking wafers/particles that were colonized in prior generations.

Self-propagation can be indicated by a jump in the value of the proxy trait over one or several generations that is then sustained over further generations. The existence of such step functions would imply that a surface-associated autocatalytic system had arisen and had then been successfully propagated to subsequent generations. Thus, it would indicate that the system possesses some kind of heritability. Depending on the proxy trait, the value that a new self-propagating system convergences upon could be either higher or lower than that seen in the control. It is worth noting that self-propagating systems might arise that are not detectable with a particular measurement, and also that two chemically distinct self-propagating systems might nonetheless converge on the same value of the proxy trait.

Finding new self-propagating chemical systems is certainly of interest, but more interesting still would be self-propagating systems that are capable of progressive evolution to new states. The features of dynamical systems that allow for transitions among quasi-stable states is an area of active research in the artificial life community, with high complexity identified as a possible promoter of open-ended evolution (Taylor et al. 2016). In a prebiotic chemical selection experiment, progressive evolution would be indicated by sequential transitions to different trait values. The simplest case would be when a self-propagating system showed steps to new states of the proxy trait, as seen for the orange and blue replicates in Fig. 3c. While it would be even more exciting to find multiple progressive evolutionary steps, the interpretation would be confounded if these happened so quickly that the pattern seen was a gradual, monotonic change over multiple generations because such a result could either be interpreted as a progressively evolving chemical system or as the emergence of a single new self-propagating system that required multiple generations to reach a stable equilibrium. Distinguishing these alternative interpretations would require looking at other proxy traits or conducting chemical analysis at different stages to see if different chemical moieties appear over time.

Assuming that self-propagating systems arise, evolvable or otherwise, the repeatability of the system would be of immediate interest. Figure 3a shows a case in which a chemically equivalent self-propagating system emerges in two of four replicates, Fig. 3b shows a case in which three different chemical systems have arisen, as indicated by convergence on different stable values of the proxy trait, and Fig. 3c illustrates a case in which all replicates converge on the same self-propagating system, but then one (purple) fails to propagate and another (orange) progresses to a second equilibrium. Documenting the degree of stochasticity in the rate of emergence of life-like chemical systems, or in the underlying chemistry that they manifest, is one of the most important insights that could be obtained from these in vitro chemical selection experiments.

### Method Extensions

There are numerous possible ways to modify the basic experiments described here, of which a few are worth mentioning. One modification to the protocol would be to include recombination. This could be accomplished most easily in particle experiments by mixing particles across replicates. In systems where multiple alternate states emerged in different replicates, it would be interesting to investigate what happens when we combine them. This can be viewed as a strategy for engineering cooperating systems of higher complexity, in cases where adaptive evolutionary steps do not happen spontaneously with sufficient frequency. It might also be desirable to recombine different systems (i.e. different particles or liquid media) that show self-propagation, but not evolvability, to see if the “hybrid” could show both phenomena.

**Fig. 4.**
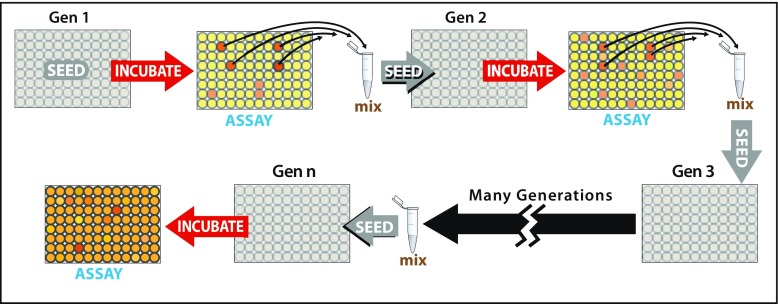
A particulate experiment with group selection. Virgin mineral particles are propagated in 96-well plates (*top row*). The plate is incubated with food and energy and then the wells are assayed, for example by quantifying the amount of redox energy used in a period of incubation. Particles are extracted from those wells showing the greatest redox energy depletion and are mixed together as “seeds” for the next generation. A small number of seed particles are added to each well of the next-generation wells, and then the cycle is repeated. If, after many such iterations, the amount of energy used per well increases then a response to selection is implied

An alternative class of modifications would deploy hierarchical selection. For example, in addition to selecting for chemical assemblages that are better at colonizing nearby mineral particles, we could also select for *populations* of mineral grains having certain target traits. Fig. 4 illustrates a potential implementation in which we expose grains in the wells of a microtiter plate to food and energy for a period of time and then assay each well for some proxy trait that is likely to indicate life-like behavior, such as the rate at which redox potential energy is dissipated. The grains from the highest-performing wells would then be recombined and distributed among all the wells of a new microtiter plate containing only virgin grains, before being exposed to energy and food for a subsequent generation. Conceptually, this approach is the one most similar to artificial ecosystem selection (Swenson et al. 2000b).

A mechanism that might contribute to continued evolution is niche construction, in which life-like chemistry substantially alters its environment (e.g. a buildup of chemicals in the food mix), thus providing raw materials or niches for additional life-like chemistries. A minimal way to incorporate this idea into the framework presented above is to serially propagate not only the particle surfaces but also a fraction of the liquid. Indeed, the ratios of new to old particles and of fresh to spent media can be independently controlled within the setups presented in Figs. 2 and 4.

### Key Variables

Chemicals in the food mix must either contain an autocatalytic set whose components can be adsorbed onto a surface, or should react spontaneously with one another to generate a larger set of chemicals that includes an autocatalytic set. Given that pretty much all the known building blocks of life are formed, albeit sometimes very inefficiently, from the simplest of precursors (CO_2_, H_2_, H_2_S, NH_3_, HCN etc.), an argument can be made that any food mix that contained a sufficient quantity of such chemicals might be sufficient. However, to increase the chances of a rapid appearance of life-like chemical systems, it seems prudent to prime the system by providing food mixtures that are enriched for potentially important, but rarely formed organic molecules. We should bear in mind that if life-like chemistry were detected using such a rich food mix, it would then be possible to repeat the experiment using more primordial starting reagents.

In choosing the chemicals to include in a food mix there are very few hard constraints, especially if one is interested in the ahistorical question of how life-like systems might arise in general. For example, there is no a priori reason to use water as the solvent (e.g., one could try formamide as suggested by Benner et al. 2012) nor to restrict oneself to molecules that might have been present on the early earth. So what approaches can be taken to guide the choice of food mixtures to start with?

There are two basic strategies one might pursue. The first would be to use prior chemical insights to select a narrow set of chemical species that are deemed likely to show the sought-after behavior. At the other extreme is the “kitchen sink” approach of including a great diversity of chemicals, using the reasoning that the probability of an autocatalytic set being present rises exponentially with the number of chemicals (Kauffman 1986; Mossel and Steel 2005). An argument against the kitchen-sink approach is that we will end up with “gunk” (Schwartz 2007) or “asphalt” (Benner et al. 2012): a complex mix of organic chemicals within which “useful” moieties are swamped by similar chemicals and polymers thereof. However, it has been noted that the gunk problem might be overcome if mineral surfaces were able to enrich for autocatalytic systems (Schwartz 2007), which is exactly what our proposed experimental approach is designed to facilitate. Furthermore, by using reasonably dilute food mixtures and an open flow system, one may hope that gunk would be constantly diluted, facilitating the emergence of autocatalytic systems that derive from a subset of species in the food mix.

Whether one uses relatively simple or complex food mixtures, a good argument can be made for starting with molecules that play important roles in cellular biochemistry. As pointed out by Strazewski (2015), since the set of molecules that life uses is demonstrably conducive to life-like chemistry, they represent a prudent starting point for prebiotic selection experiments. If life-like chemical systems emerged successfully, there would always be the option of later trying other food mixtures to better understand the chemical features that allow the formation of surface-associated, self-propagating systems. Nonetheless, while we would advocate starting with a food mix compatible with conventional biochemistry, there might be value to at least spiking the mix with some molecules that do not play important roles in living cells, as well as chiral variants that are absent from life. One advantage of including these non-standard building blocks is that it might make it easier to show that a chemical assemblage arose de novo rather than being generated by contaminating cells.

Abundant physical theory shows that the emergence of order in living systems requires far from equilibrium thermodynamics (Pross and Pascal 2013). Thus, in addition to adding a constant supply of food, some replenishing source of energy needs to be provided. While Miller-Urey experiments typically used intense physical energy sources, such as heat, light, electrical discharges, or sound (reviewed by Keosian 1968), we believe that it would be more promising to focus on chemical energy. The constructive, thermodynamically uphill chemical reactions that characterize cellular life can sometimes be driven by physical energy sources (most commonly light, but rarely mechanical forces), but are much more often driven by coupling of those reactions with other thermodynamically downhill redox reactions.

Chemical energy could be provided in organic or inorganic forms. Most models of early metabolism are driven by redox reactions, often beginning by reduction of carbon dioxide by hydrogen (Wächtershäuser 1988), which is an appealing hypothesis because such a reaction dissipates energy by forming complex organic molecules (Morowitz and Smith 2007). However, experiments need not be restricted to putatively primordial energy sources. One could try any number of high-energy organic compounds, for example nucleotide triphosphates or organic anhydrides (Strazewski 2015). Likewise, there are diverse possible ways to provide redox potential energy using inorganic salts, for example including mixtures of ions that are well out of thermodynamic equilibrium but react slowly in aqueous solutions. Indeed, a case can be made for including multiple organic and inorganic energy sources in a combined food/energy mix because chemical diversity could maximize the chances of an autocatalytic cycle being present (Kauffman 1986; Mossel and Steel 2005) and, if present, the chances of them persisting in the face of parasitic side reactions (Virgo et al. 2013).

The theory of neighborhood selection requires the existence of a surface upon which chemicals can adhere and perhaps also engage in catalysis and metabolic channeling. However, there is nothing that requires a particular topology of surface – with possibilities ranging from a flat surface, to a porous mineral, or a colloidal matrix. Furthermore, it is not obvious that the surface need be rigid. For example, the outside of lipsomes are possible surfaces for initial colonization (Strazewski 2015). So what surfaces would be best to try first?

There is no compelling reason to think that the kind of surface upon which life actually arose (assuming it did arise on a surface at all) would be the only kind of surface upon which life-like chemistry *could* arise. Nonetheless, since life-like chemistry did demonstrably arise during the origin of life, it seems prudent to focus on surfaces upon which life might indeed have arisen. Several minerals have been proposed as possible players in the origin of life (Hazen and Sverjensky 2010), including metal sulfides, such as iron pyrite (Wächtershäuser 1988; Huber and Wächtershäuser 1998), and various clays, long noted for their surface adhesion and catalytic properties (Smith-Cairns 1966; Ponnamperuma et al. 1982; Hanczyc et al. 2003). However, in the long run it would surely be desirable to try many alternative surfaces in the search for ones especially conducive to the rapid appearance of life-like chemical consortia.

The most important aspect of the physical environment (temperature, pressure, light, ionic strength, and pH) for such prebiotic selection experiments is that it be stable or regularly cycling, since this would allow us to attribute any systematic changes to self-propagation. Apart from the presumption that life on Earth arose within liquid water, there is little agreement as to the physical setting of life’s emergence. For example, some have favored geothermal surface pools, which is to say warm, low pressure, high light, and low ionic strength environments (DeGuzman et al. 2014), whereas others lean towards deep sea hydrothermal vents, which is to say high heat, high pressure, low light, higher ionic strength, and either high or low pH, depending on the kind of vent (Wächtershäuser 1990; Huber and Wächtershäuser 1998; Martin and Russell 2007; Herschy et al. 2014). Ultimately, it would seem prudent to try a wide diversity of physical environments. However, there is nothing to argue against selecting experimentally convenient situations at the outset, such as room temperature, low light, atmospheric pressure.

### Controls and Caveats

As discussed earlier, we need appropriate controls to correct for the fact that, however hard we may attempt to control them, conditions are unlikely to remain constant over many generations of selection. For example, the food mix could change spontaneously or show batch-to-batch variation. Moreover, even if a signal of self-propagation is seen in experimental but not control runs, we would need to ensure that the results are not due to microbial contamination. While not technically a “false” positive, since living cells manifest the self-same properties that we are looking for, discovery that bacteria can propagate and evolve adaptively would hardly be interesting! Fortunately, we know what cellular life looks like under the microscope and know how to detect it, for example by quantifying DNA. Thus, if a response to selection were observed one could readily use microscopic and chemical or molecular biological analyses to see if conventional, nucleic-acid based life is present.

Assuming that the practical issues are suitably dealt with, could a systematic response to selection be obtained even in the absence of truly life-like chemistry? For example, could an apparent response to selection be due to a nucleated crystal-growth phenomenon devoid of interesting chemical activities, such as redox chemistry or organic polymerization? While this cannot be ruled out, it is hard to imagine a crystallization process that could systematically get quicker over multiple generations. Furthermore, even if this were the case, the fact that the system was capable of evolving towards improved self-propagation is interesting and could, potentially, serve as a foundation upon which more complex chemistries could evolve later.

Another possible critique of the proposed experiments is that imposing strong artificial selection for a proxy trait (such a redox energy use) would not establish that *natural* selection on surface-bound chemical assemblages could have driven the emergence of life on earth. After all, the argument would go, humans were not around then to impose artificial selection making this experiment formally irrelevant to the natural origin of life. However, in the same way that Darwin used artificial selection of crops and domesticated animals as an instructive analog for natural selection, observing a response to artificial selection for a proxy trait would be very helpful in showing that the capacity to respond to selection can arise in spontaneously-formed chemical systems. Thus, while more naturalistic experiments would certainly be worth conducting, we would consider any response to selection for a proxy trait, however artificial, to be an important step forward in understanding the emergence of life.

## Conclusions and Prospects

Recent advances, including an ever narrowing time-window for the appearance of cellular life, the development of concepts of inheritance that are not tied to digital polymer-encoding systems, and the ideas of surface metabolism and neighborhood selection, suggest that the origin of life may be much easier than previously appreciated. As argued in this paper, these new insights suggest various simple experiments that have the potential to document life-like, surface-bound chemical assemblages in the time frame of laboratory science.

It will no doubt occur to the reader that the experiments proposed are high-risk, in the sense that negative results are likely. This poses the question of what, if anything, would be gained through such failures. Showing, through systematic exploration of many chemical mixtures, that combining complex chemical building blocks and ample free energy is, by itself, insufficient to result in self-propagation would be highly informative in suggesting the need to consider other mechanisms to explain the origin of life. Similarly, finding that self-propagation is relatively generic, but that these self-propagating systems are rarely evolvable, can shape our perspective, for example by focusing efforts on how higher-order organization can emerge through cooperation of self-propagating systems. Finally, discovery of even a single system capable of a few adaptive evolutionary steps would certainly be an important contribution that could launch efforts to further understand what phenomena permit or prevent open-ended evolution. Thus, there are various ways in which failure or partial success could be valuable contributions.

What we have described is not a single experiment but an experimental concept based on the search for new life-like chemical systems that show evidence of self-propagation and adaptive evolution. There are an almost infinite number of actual experiments that would fit this paradigm, and we hope that scientists from diverse disciplinary backgrounds will use the principles described here to design concrete experiments along these lines. It could be that very specific conditions are needed. In that case, there is virtue in a large community of researchers trying such experiments so as to collectively explore a broad range of conditions. Alternatively, it could be that life-like chemistry arises quite easily under almost any conditions that meet basic constraints such as sufficient energy and appropriately diverse food mixtures. In that case, whoever does the experiments first will have the pleasure of validating this conceptual framework, but even then the community at large will have the opportunity to address important questions such as the repeatability and predictability of chemical ecosystem evolution. We, therefore, sincerely hope that this conceptual paper stimulates other empiricists to develop and implement diverse prebiotic selection experiments.
